# Resilience of the slow component in timescale-separated synchronized oscillators

**DOI:** 10.3389/fnetp.2024.1399352

**Published:** 2024-06-19

**Authors:** Melvyn Tyloo

**Affiliations:** Theoretical Division and Center for Nonlinear Studies (CNLS), Los Alamos National Laboratory, Los Alamos, NM, United States

**Keywords:** synchronization & phase locking, timescale separation, stochastic and deterministic stability, coupled oscillators, network physiology, complex networks

## Abstract

Physiological networks are usually made of a large number of biological oscillators evolving on a multitude of different timescales. Phase oscillators are particularly useful in the modelling of the synchronization dynamics of such systems. If the coupling is strong enough compared to the heterogeneity of the internal parameters, synchronized states might emerge where phase oscillators start to behave coherently. Here, we focus on the case where synchronized oscillators are divided into a fast and a slow component so that the two subsets evolve on separated timescales. We assess the resilience of the slow component by, first, reducing the dynamics of the fast one using Mori-Zwanzig formalism. Second, we evaluate the variance of the phase deviations when the oscillators in the two components are subject to noise with possibly distinct correlation times. From the general expression for the variance, we consider specific network structures and show how the noise transmission between the fast and slow components is affected. Interestingly, we find that oscillators that are among the most robust when there is only a single timescale, might become the most vulnerable when the system undergoes a timescale separation. We also find that layered networks seem to be insensitive to such timescale separations.

## 1 Introduction

The synchronization dynamics of coupled phase oscillators finds numerous applications ranging from Josephson junctions and electrical power grids to physiological networks (Wiesenfeld et al., 1998; Pikovsky et al., 2003; Acebrón et al., 2005; Strogatz, 2014; Stiefel and Ermentrout, 2016; Ji et al., 2023). The collective behavior displayed by these systems is made possible by the interplay between the internal parameters of the individual dynamical units and the interaction coupling their degrees of freedom (Winfree, 1967; Kuramoto, 1975; Kuramoto, 1984; Strogatz, 2000). Due to the nonlinear nature of the coupling together with the complex network topology of the interaction, multiple synchronized states might exist for the same parameters and might be visited by the system due to perturbations or noise (Kramers, 1940; Dykman, 1990; DeVille, 2012; Rodrigues et al., 2016). Importantly, synchronization is not always a desirable feature. For example, in electrical power grids, a synchronous operational state ensures the good functioning and distribution of power (Blaabjerg et al., 2006; Machowski et al., 2008; Dörfler et al., 2013). The answer is less binary in physiological systems. Indeed, synchronization is of primal importance for some cognitive processes in the brain ensuring an adequate level of communication between neuronal groups (Fries, 2005). Also, synchronized dynamics emerge in healthy neuronal systems during sleep (González et al., 2023). Thus, a lack of synchronization might result in some impairment of physiological systems. However, direct connections have been drawn between the excess of synchronization in some neuronal groups and brain diseases (Uhlhaas and Singer, 2006; Popovych and Tass, 2014). Therefore, the synchronization dynamics as well as its resilience to external perturbations are topics of primal importance in order to better understand the interplay between synchronized groups of dynamical units.

Different types of synchronization might occur. For example, all the phases may converge to the same global value, which is usually referred to as *phase synchronization*. Another type of synchronization happens when the frequencies of all the oscillators converge to a common global value, which is referred to as *phase-locked state*, with time-independent phase differences. Perturbations of these synchronized states can take a great variety of forms such as external input signals injected into some internal parameters or noisy environments (DeVille, 2012; Hindes and Myers, 2015; Schäfer et al., 2017; Hindes and Schwartz, 2018; Ronellenfitsch et al., 2018; Tyloo et al., 2018; Halekotte and Feudel, 2020; Tyloo, 2022a), interruption of the interaction between some oscillators due to local failures (Soltan et al., 2017; Delabays et al., 2022), alteration of the dynamics of some units (Wang and Wang, 2019; Tyloo, 2023). Here, we are interested in networks of phase oscillators in a phase-locked state where, due to some damage to a subset of oscillators or simply because of their intrinsic characteristics, two separate timescales of the dynamics exist so that the system is divided into a fast and a slow component. This kind of timescale separation might occur for example, in the human physiological system thanks to the wide range of timescales reported (Gao et al., 2020). In such a scenario, the fast oscillators adapt to any input signal quickly compared to the ones in the slow component. Therefore, the input signals into the oscillators belonging to the fast component are transmitted differently to those in the slow component compared to the case where all oscillators evolve on the same timescale. Such timescale separation in systems of coupled phase oscillators have been used in the modelling of power systems (Kokotovic et al., 1980) and synchronization dynamics of Kuramoto oscillators with attractive and repulsive couplings (Kirillov et al., 2020). As a paradigmatic model to investigate synchronization, we use Kuramoto oscillators, but the framework presented here applies more generally to coupled dynamical systems evolving close to a stable fixed point. We consider time-correlated noisy inputs as in many relevant situations, dynamical systems are constantly pushed away from their synchronized fixed point by ambient noisy conditions (van Kampen, 1976). The resilience of the system to such perturbations can be assessed in various ways. One can estimate the size of the basin of attraction (Wiley et al., 2006; Menck et al., 2013), or evaluate the amplitude of the small fluctuations or the escape rate of large fluctuations (Tyloo, 2022b; Hindes et al., 2023). In this manuscript, we assess the resilience of the slow component in the small fluctuation regime by quantifying the phase deviations from the synchronized state. This is important as it clarifies how the features of the dynamical system affect its robustness to noise when coupled oscillators evolve over multiple timescales. Within the assumption of small fluctuation, we investigate the linear response of the system around a stable fixed point. We first account for the timescale separation applying Mori-Zwanzig formalism (Mori, 1965; Zwanzig, 1973) to the slow and fast components. This leads to a reduced dynamics of the oscillators which is equivalent to a Kron reduction of the Jacobian matrix (Kron, 1939; Dörfler and Bullo, 2012). The latter elucidate how the inputs in the fast component are transmitted to the slow one. Then, solving the linear system, we calculate the variance of the phase deviations in the slow component when time-correlated noise inputs with distinct typical correlation times are applied in each component. We show how the amplitude of the excursion essentially depends on the characteristics of the noise, as well as the system properties through the spectrum of its reduced Jacobian. In some specific settings, we are able to further predict the transmission of the noise from the fast to the slow component based on the properties of the oscillators in the fast component as well as the inter-component coupling structure. In particular, we find that some oscillators having smaller variance when there is no timescale separation, might become the ones with larger variance when there is a timescale separation, if they are well connected to the fast component. Also, when the slow and fast components are defined on a layered network, the variance is mostly insensitive to the timescale separation.

In Section 2, we introduce the model of Kuramoto oscillators with timescale separation and apply Mori-Zwanzig formalism to obtain a reduced dynamics for the slow component. In the same section, we then calculate the variance of the degrees of freedom of the oscillators in the slow component subject to time-correlated noise. In Section 3, we numerically confirm and illustrate the theory on various network structures. The conclusions are given in Section 4.

## 2 Timescale separation

Here, we first introduce the model of noisy phase oscillators we investigate and how the timescale separation is mathematically taken into account. Then, we describe the near-equilibrium dynamics which is captured by the linear response of the system, and apply Mori-Zwanzig formalism to obtain the time-evolution in the slow component. This enables us to calculate the moments of the phase deviations of each oscillator in the slow component. Eventually, we consider the strong coupling limit where the expression for the variance of the phase deviation can be explicitly calculated for specific network structures.

### 2.1 Networks of phase oscillators

We are interested in the situation where, due to an external perturbation or change in the environment, the intrinsic timescales of the individual oscillators separate into a fast and a slow subsystem. We consider a set of *N* oscillators each with a compact phase degree of freedom *θ*_*i*_ ∈ (−*π*, *π*] whose time-evolution is governed by the set of coupled differential equations (Kuramoto, 1975),

$$\begin{array}{c} d_{i}\;{\dot{\theta }}_{i}=\omega_{i}-\overset{N}{\underset{j=1}{\sum }}b_{ij}\;\sin\left(\theta_{i}-\theta_{j}\right)+\eta_{i}, \end{array}$$ (1)

for *i* = 1, … *N*. The natural frequency of the *i*th oscillator is denoted *ω*_*i*_, the structure of the coupling network is given by elements *b*_*ij*_ of the adjacency matrix (Newman, 2018). Ambient noise is modelled at the *i*th oscillator by *η*_*i*_ and is taken as a time-correlated noise, uncorrelated in space, i.e., $\left\langle \eta_{i}\left(t\right)\eta_{j}\left(t^{\prime }\right)\right\rangle =\eta_{0,i}^{2}\delta_{ij}\exp\left[-|t-t^{\prime }|/\tau_{i}\right]$ , where *τ*_*i*_ is the typical correlation time of the noise at the *i*th oscillator. The non-negative parameters *d*_*i*_’s define the individual timescale of each oscillator. Removing the noise term, Eq. 1 may have multiple stable fixed points of the dynamics which essentially depend on the coupling topology and strength as well as the distribution of natural frequencies. Below, we assume that such a stable fixed point $\left\{\theta_{i}^{*}\right\}$ exists and that the noise term is small enough such that the dynamics remains inside the initial basin of attraction. In the present scenario, we assume that we have two sets of oscillators that we denote F and S , respectively with $N_{F}$ and $N_{S}$ oscillators, such that

$$d_{i}=\left\{\begin{array}{cc} \bar{d}\; & i\in F\\ \underline{d}\; & i\in S \end{array}\right.$$ (2)

with $\bar{d}\ll \underline{d}$ . The latter means that oscillators belonging to S have a much slower intrinsic timescale than those belonging to F . In the following, we focus on the dynamics of the oscillators in the slow component. Within the assumption of timescale separation, one can rewrite Eq. 1 as,

$$\begin{array}{cc} \;{\dot{\theta }}_{i} & =\omega_{i}-\overset{N}{\underset{j=1}{\sum }}b_{ij}\;\sin\left(\theta_{i}-\theta_{j}\right)+\eta_{i},\;i\in S\\ \epsilon \;{\dot{\theta }}_{i} & =\omega_{i}-\overset{N}{\underset{j=1}{\sum }}b_{ij}\;\sin\left(\theta_{i}-\theta_{j}\right)+\eta_{i},\;i\in F, \end{array}$$ (3)

where we defined $\underline{d}/\bar{d}=\epsilon^{-1}$ and, without loss of generality, set $\underline{d}$ to unity. In the limit *ϵ* → 0, the oscillators within F instantaneously adapt their phases. In the next section, we consider the dynamical system Eq. 3 in the vicinity of a stable fixed point and perform a singular perturbation analysis using Mori-Zwanzig formalism.

### 2.2 Near-equilibrium and reduced dynamics

Even though we consider Kuramoto oscillators, the following approach applies in general to coupled dynamical systems that have a stable fixed point around which they evolve and where linearization is valid. To analyze the resilience of the slow component, we consider the dynamics of the system close to a fixed point $\left\{\theta_{i}^{*}\right\}$ . In particular, we are interested in the time-evolution of the phase deviations $x_{i}\left(t\right)=\theta_{i}\left(t\right)-\theta_{i}^{*}$ for i∈S and $y_{i}\left(t\right)=\theta_{i}\left(t\right)-\theta_{i}^{*}$ for i∈F whose dynamics at the first order reads,

$$\left[\begin{array}{c} \dot{x}\\ \epsilon \;\dot{y} \end{array}\right]=\left[\begin{array}{cc} J_{SS} & J_{SF}\\ J_{FS} & J_{FF} \end{array}\right]\left[\begin{array}{c} x\\ y \end{array}\right]+\left[\begin{array}{c} \eta_{S}\\ \eta_{F} \end{array}\right]$$ (4)

where we defined the matrix

$$J_{ij}=\left\{\begin{array}{cc} b_{ij}\cos\left(\theta_{i}^{*}-\theta_{j}^{*}\right)\; & i\neq j\\ -\overset{N}{\underset{k=1}{\sum }}b_{ik}\cos\left(\theta_{i}^{*}-\theta_{k}^{*}\right)\; & i=j, \end{array}\right.$$ (5)

which is the Jacobian of the system and is a Laplacian matrix when phase differences are between $-\frac{\pi }{2}$ and $\frac{\pi }{2}$ . Using Mori-Zwanzig formalism (Mori, 1965; Zwanzig, 1973) with **x** and **y** being respectively the resolved and unresolved variables (see Supplementary Material Appendix A), one can express the first row of Eq. 4 as,

$$\begin{array}{ll} {\dot{x}}_{i} & =\overset{N_{S}}{\underset{j=1}{\sum }}{J_{SS}}_{ij}\;x_{j}+{\eta_{S}}_{i}+\overset{N_{F}}{\underset{\alpha =1}{\sum }}\int_{0}^{t}\epsilon^{-1}e^{\nu_{\alpha }\epsilon^{-1}\left(t-t^{\prime }\right)}\\ & \;\times \overset{N_{F}}{\underset{k,m=1}{\sum }}\overset{N_{S}}{\underset{l=1}{\sum }}{J_{FS}}_{kl}x_{l}\left(t^{\prime }\right)w_{\alpha ,k}{J_{SF}}_{im}w_{\alpha ,m}\;dt^{\prime }\\ + & \overset{N_{F}}{\underset{\alpha =1}{\sum }}\int_{0}^{t}\epsilon^{-1}e^{\nu_{\alpha }\epsilon^{-1}\left(t-t^{\prime }\right)}\overset{N_{F}}{\underset{k,m=1}{\sum }}{\eta_{F}}_{k}\left(t^{\prime }\right)w_{\alpha ,k}{J_{SF}}_{im}w_{\alpha ,m}\;dt^{\prime }, \end{array}$$ (6)

where we denoted **w**_*α*_ the eigenvectors of $J_{FF}$ with corresponding eigenvalues *ν*_*α*_ < 0. In Eq. 6, the first two terms on the right-hand side are Markovian, i.e., they do not depend on the history of the trajectories, while the other ones have memory and thus do depend on the history. We are interested in the time-evolution of the slow component **x** when there is a timescale separation, i.e., *ϵ* → 0. Taking the latter limit in Eq. 6, and using $\lim_{\varphi \to \infty }\int_{0}^{t}\varphi e^{\varphi \;t^{\prime }}f\left(t^{\prime }\right)dt^{\prime }=f\left(0\right)$ (see Supplementary Material Appendix B), and the identity $J_{FF}^{-1}=\sum_{\alpha }\nu_{\alpha }^{-1}w_{\alpha }w_{\alpha }^{⊤}$ , yields in a matrix form,

$$\begin{array}{ll} \dot{x} & =J_{SS}\;x-J_{SF}\;J_{FF}^{-1}\;J_{FS}\;x+\eta_{S}-J_{SF}\;J_{FF}^{-1}\;\eta_{F}\\ & =J_{\text{red}}\;x+\xi , \end{array}$$ (7)

where in the second line we defined the reduced Jacobian $J_{\text{red}}=J_{SS}-J_{SF}\;J_{FF}^{-1}\;J_{FS}$ , and denoted the noise term as $\xi =\eta_{S}-J_{SF}\;J_{FF}^{-1}\;\eta_{F}$ . It is interesting to note that the reduced dynamics given by Eq. 7 can be obtained by a Kron reduction (Kron, 1939; Dörfler and Bullo, 2012) of the fast component of the system (see (Tyloo et al., 2023) for an example). The dynamics of the slow component is then governed by Eq. 7 where the effective noise at the *i*th oscillator is a combination of the noise at the *i*th oscillator with a superposition of the noise inputs at oscillators belonging to the fast component. For undirected coupling as we consider in the following, one has that $J_{FS}=J_{SF}^{⊤}$ . The linear system Eq. 7 can be solved by expanding over the eigenmodes of **J**_red_ denoted **u**_*α*_, with corresponding eigenvalues *λ*_*α*_, $\alpha =1,\ldots N_{S}$ , i.e., **x**(*t*) = *∑*_*α*_ *c*_*α*_(*t*)**u**_*α*_. As **J**_red_ is also the negative of a Laplacian matrix, one has that $0=\lambda_{1}>\ldots \geq \lambda_{N_{S}}$ with $u_{1,i}=1/\sqrt{N_{S}}$ . The expansion coefficients satisfy the uncoupled differential equations,

$${\dot{c}}_{\alpha }=\lambda_{\alpha }c_{\alpha }+\xi \cdot u_{\alpha },\;\alpha =1,\ldots ,N_{S}.$$ (8)

Assuming a vanishing initial condition, the general solution to Eq. 7 is given by,

$$\begin{array}{ll} x_{i}\left(t\right) & =\overset{N_{S}}{\underset{\alpha =2}{\sum }}\int_{0}^{t}\exp\left[\lambda_{\alpha }\left(t-t^{\prime }\right)\right]u_{\alpha }\cdot \xi \left(t^{\prime }\right)\;dt^{\prime }u_{\alpha ,i}\\ & +\frac{t}{N_{S}}\overset{N_{S}}{\underset{j=1}{\sum }}\xi_{j}, \end{array}$$ (9)

for $i=1,\ldots N_{S}$ . One remarks that, if $\sum_{j=1}^{N_{S}}\xi_{j}\neq 0$ , then all the oscillators in the slow component drift simultaneously together in time, i.e., along **u**_1_. Such a homogeneous overall shift in the phases of all the oscillators in the slow component does not change the system. Indeed, this is due to the global rotational symmetry of the original system Eq. 3, and the timescale separation. Accordingly, in the following, all the averages over time or noise sequences, denoted ⟨.⟩, correspond to contributions orthogonal to **u**_1_. The expression Eq. 9 can be used directly to calculate the moments of the phase deviations.

### 2.3 Fluctuations from the synchronized state

Various characteristics of the response can be used to determine the resilience of the coupled oscillators. When subject to stochastic inputs, a natural choice is to evaluate the magnitude of the deviations from the synchronized fixed point by calculating the variance of the phase deviations. Here, we consider time-correlated noise of the form given below Eq. 1, with $\tau_{i}=\tau_{S}$ if i∈S and $\tau_{i}=\tau_{F}$ if i∈F , where $\tau_{S}$ , $\tau_{F}$ are the typical correlation times respectively, in the slow and fast component. This noise translates into the reduced noise as $\left\langle \xi_{j}\left(t_{1}\right)\xi_{k}\left(t_{2}\right)\right\rangle =\eta_{0}^{2}\;\delta_{jk}\exp\left(-|t_{1}-t_{2}|/\tau_{S}\right)+\eta_{0}^{2}{\left[J_{SF}J_{FF}^{-2}J_{FS}\right]}_{jk}\exp\left(-|t_{1}-t_{2}|/\tau_{F}\right)$ . The variance of the phase deviations in the slow component is defined as $\left\langle {\left(x_{i}-N_{S}^{-1}\sum_{j\in S}x_{j}\right)}^{2}\right\rangle$ and simply denoted as $\left\langle x_{i}^{2}\right\rangle$ in the rest of this manuscript to shorten the notation. It is calculated from Eq. 9 and reads in the long time limit (see Supplementary Material Appendix C),

$$\begin{array}{ll} \left\langle x_{i}^{2}\right\rangle & =\eta_{0,S}^{2}\overset{N_{S}}{\underset{\alpha =2}{\sum }}\frac{u_{\alpha ,i}^{2}}{\lambda_{\alpha }\left(\lambda_{\alpha }-\tau_{S}^{-1}\right)}\\ +\eta_{0,F}^{2} & \overset{N_{S}}{\underset{\alpha ,\beta =2}{\sum }}\frac{\left(\lambda_{\alpha }+\lambda_{\beta }-2\tau_{F}^{-1}\right)\Gamma_{\alpha \beta }\;u_{\alpha ,i}u_{\beta ,i}}{\left(\lambda_{\alpha }+\lambda_{\beta }\right)\left(\tau_{F}^{-1}-\lambda_{\alpha }\right)\left(\tau_{F}^{-1}-\lambda_{\beta }\right)}, \end{array}$$ (10)

with the scalar $\Gamma_{\alpha \beta }=u_{\alpha }^{⊤}J_{SF}J_{FF}^{-2}J_{FS}u_{\beta }$ . In Eq. 10, we set the standard deviation of the ambient noise in the slow and fast components respectively, to $\eta_{0,S}$ and $\eta_{0,F}$ . We also set distinct homogeneous correlation times for the noise in each component as $\tau_{i}=\tau_{S}$ for i∈S and $\tau_{i}=\tau_{F}$ for i∈F . While the contribution to the variance from the additive noise in the slow component is essentially given by the position of the oscillators on the slowest eigenmodes, the effect of the noise coming from the fast component involves combinations of eigenmodes. The precise combination depends on the effective reduced dynamics through Γ_*αβ*_. The shortest timescale in the system is set by the oscillators belonging to F . However, by tuning the correlation time of the noise $\tau =\tau_{F}=\tau_{S}$ , one can investigate the regimes where the $\lambda_{N_{S}}\tau \ll 1$ and *λ*_2_ *τ* ≫ 1. Indeed, in the limit where the noise correlation time is shorter than the timescales of the slow component and the same in both components, the variance becomes,

$$\left\langle x_{i}^{2}\right\rangle =\eta_{0,S}^{2}\;\tau \overset{N_{S}}{\underset{\alpha =2}{\sum }}\frac{u_{\alpha ,i}^{2}}{\left(-\lambda_{\alpha }\right)}+\eta_{0,F}^{2}\;\tau \overset{N_{S}}{\underset{\alpha ,\beta =2}{\sum }}\frac{2\;\Gamma_{\alpha \beta }\;u_{\alpha ,i}u_{\beta ,i}}{\left(-\lambda_{\alpha }-\lambda_{\beta }\right)}.$$ (11)

In the other limit where the noise correlation time is the longest timescale, one has,

$$\left\langle x_{i}^{2}\right\rangle =\eta_{0,S}^{2}\;\overset{N_{S}}{\underset{\alpha =2}{\sum }}\frac{u_{\alpha ,i}^{2}}{\lambda_{\alpha }^{2}}+\eta_{0,F}^{2}\;\overset{N_{S}}{\underset{\alpha ,\beta =2}{\sum }}\frac{\Gamma_{\alpha \beta }\;u_{\alpha ,i}u_{\beta ,i}}{\lambda_{\alpha }\lambda_{\beta }}.$$ (12)

Comparing the two limiting cases Eqs 11, 12, one remarks that in both variances, a significant contribution might come from the slowest eigenmodes. Note also that Eq. 10 is more generally valid in the case where $\tau_{F}$ and $\tau_{S}$ are different.

In the Supplementary Material Appendix D, we give the variance of the phases when there is no timescale separation.

To obtain more insights into the contribution from the fast component, let us consider specific situations in the strong coupling limit.

### 2.4 Strong coupling limit

In the strong coupling limit, one has $|\theta_{i}^{*}-\theta_{j}^{*}|\ll 1\;\forall i,j$ , so that one can approximate the Jacobian Eq. 5 as,

$$J_{ij}=\left\{\begin{array}{cc} b_{ij}\; & i\neq j\\ -\overset{N}{\underset{k=1}{\sum }}b_{ik}\; & i=j. \end{array}\right.$$ (13)

Within this coupling limit and some other assumptions that are specified below, one can further consider network structures that give more insights about Eq. 10.

#### 2.4.1 Disconnected oscillators in the fast component

**FIGURE 1:**
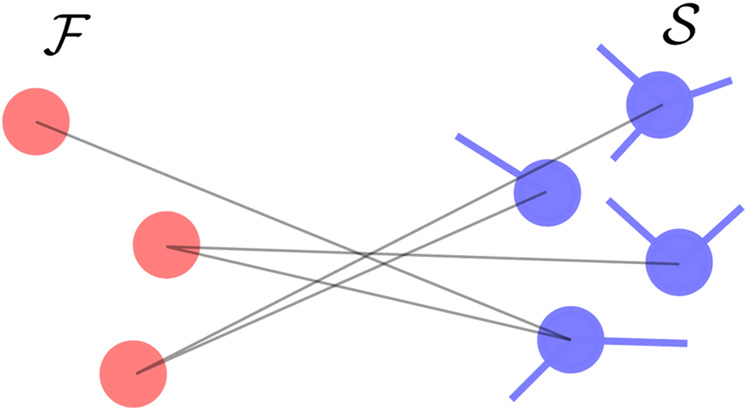
Connectivity where oscillators in the fast component are disconnected. The fast oscillators are shown in red while the slow oscillators are shown in blue.

In the simple scenario where only a single oscillator *l* belongs to the fast component while all the others are in the slow one, $J_{FF}$ is a scalar such that $J_{FF}^{-2}=k_{l}^{-2}$ is the inverse of the squared weighted degree of the fast oscillator indexed by *l*. Therefore, one has $\Gamma_{\alpha \beta }=\left(\sum_{j\in N\left(l\right)}u_{\alpha ,j}b_{lj}\right)\left(\sum_{k\in N\left(l\right)}u_{\beta ,k}b_{lk}\right)/k_{l}^{2}$ , where N(l) is the set of oscillators in the slow component connected to the fast oscillator *l*. The contribution from the second term in Eq. 10 therefore depends on the location of the oscillators on the slowest eigenmodes of the reduced Jacobian. This situation easily generalizes to the case of multiple oscillators in the fast component that are not connected as shown in Figure 1. One then has $\Gamma_{\alpha \beta }=\sum_{l\in F}\left(\sum_{j\in N\left(l\right)}u_{\alpha ,j}b_{lj}\right)\left(\sum_{k\in N\left(l\right)}u_{\beta ,k}b_{lk}\right)/k_{l}^{2}$ , where we took the sum over all the oscillators in the fast component. Due to the dependence of Γ_*αβ*_ on $k_{l}^{2}$ and *b*_*l*_, we expect the contribution to the variance from the fast component to be rather small in general.

#### 2.4.2 All-to-all coupling from fast to slow component

**FIGURE 2:**
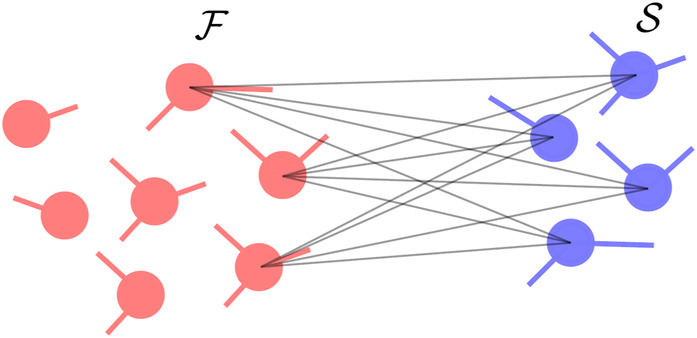
Connectivity where oscillators in the fast component with inter-component connections are connected to all the oscillators in the slow component (see inset). The fast oscillators are shown in red while the slow oscillators are shown in blue.

When the oscillators in the fast component that have inter-component connections are connected to all the oscillators in the slow component with homogeneous coupling, i.e., $b_{lj}=b_{l}>0\;\forall j\in N\left(l\right)=S$ , with l∈F (see Figure 2), the second term in Eq. 10 vanishes. Indeed, in such a situation, the columns of the matrix $J_{SF}=J_{FS}^{⊤}$ corresponding to the inter-component coupling are full of *b_l_*’s and as **u**_1_⋅**u**_*α*_ = *δ*_1*α*_ by orthogonality of the eigenmodes, one has that Γ_*αβ*_ = 0 for all $\alpha ,\beta =2,\ldots ,N_{S}$ . Intuitively, if the signal from one oscillator in the fast component is transmitted with the same strength to all the oscillators in the slow one, then it will result in an overall phase shift without affecting the fixed point. However, if the coupling is not homogeneous between the slow and fast components, the variance will be different from zero.

#### 2.4.3 All-to-all from slow to fast component

**FIGURE 3:**
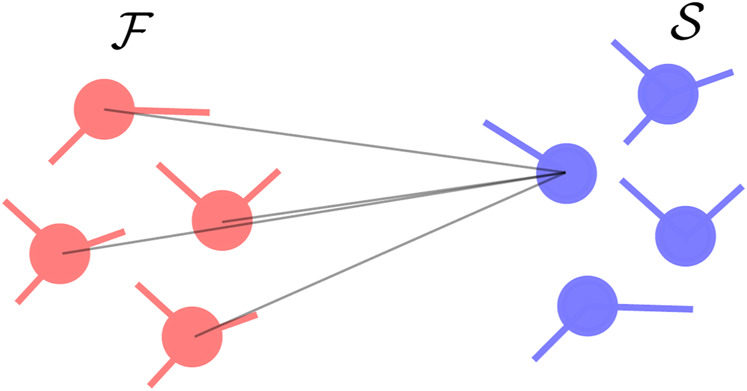
Connectivity where a single oscillator in the slow component is connected to all the oscillators in the fast one. The fast oscillators are shown in red while the slow oscillators are shown in blue.

In the opposite case where only a single oscillator in the slow component is homogeneously connected to all the oscillators in the fast one (see Figure 3), the intra-component structure of the coupling within the fast component does not influence the propagation of the noise. Indeed, if $b_{lj}=b_{l}>0\;\forall j\in M\left(l\right)=F$ where here M(l) is the set of oscillators in the fast component connected to the *l*th oscillator in the slow component, one has $\Gamma_{\alpha \beta }=u_{\alpha ,l}u_{\beta ,l}N_{F}$ . Therefore, only the size of the fast component and not the intra-component network structure of the oscillators influence the variance in the slow one. This can be generalized to the case where multiple oscillators in slow component are connected to all the ones in the fast component. One then has, $\Gamma_{\alpha \beta }=\underset{k,l\in N\left(F\right)}{\sum }u_{\alpha ,k}u_{\beta ,l}m^{-2}N_{F}$ with N(F) the set of oscillators in the slow component with all-to-all connections to the fast one whose size is denoted m. Here, we assumed that the inter-component coupling strength is homogeneous.

#### 2.4.4 Layered networks

**FIGURE 4:**
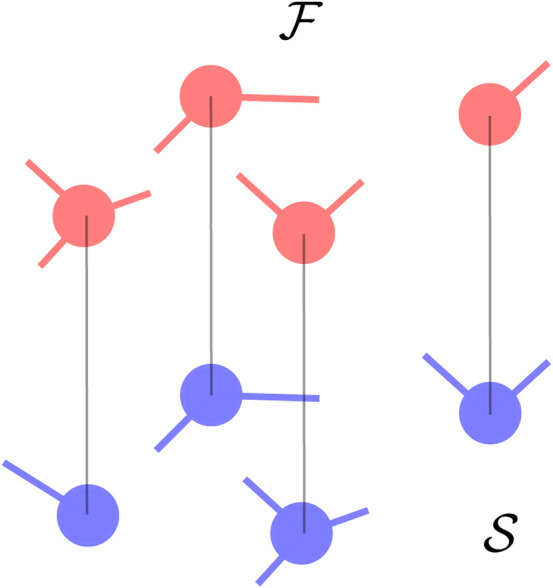
Connectivity in layers where the fast oscillators are shown in red in the top layer while the slow oscillators are shown in blue in the bottom layer. Each oscillator in one of the layers can be connected only to a single oscillator in the other layer.

An interesting case arises when oscillators are connected on layered networks so that the fast component is on one layer, the slow one on another layer, and the two layers are connected together. In the specific scenario where each fast oscillator is connected to a single distinct oscillator in the slow component and the number of units in the layers is the same, one has that $J_{SF}$ is a diagonal matrix (up to a permutation of the oscillators indices). This is illustrated in Figure 4. If one further assumes that the inter-layer coupling is homogeneous, i.e., $J_{SF}=\tilde{b}\;I$ is a multiple of the identity matrix, one has that the eigenbases of **J**_red_ and $J_{SS}=J_{FF}$ satisfy the following relations.

$$J_{\text{red}}u_{\alpha }=\lambda_{\alpha }u_{\alpha }=\left(J_{SS}-{\tilde{b}}^{2}{J_{SS}}^{-1}\right)u_{\alpha },$$ (14)

$$\left(J_{SS}-{\tilde{b}}^{2}{J_{SS}}^{-1}\right)v_{\beta }=\left(\mu_{\beta }-{\tilde{b}}^{2}\mu_{\beta }^{-1}\right)v_{\beta }=J_{\text{red}}v_{\beta },$$ (15)

with $\alpha ,\beta =1,\ldots ,N_{S}$ and where the eigenmodes of $J_{SS}$ are denoted **v**_*β*_ with corresponding eigenvalues *μ*_*β*_. Noticing that $\left(\mu_{\beta }-{\tilde{b}}^{2}\;\mu_{\beta }^{-1}\right)$ is a monotonically increasing function of *μ*_*β*_, one has a one-to-one correspondence between the eigenmodes of **J**_red_ and $J_{SS}$ such that **u**_*α*_ = **v**_*α*_ and $\lambda_{\alpha }=\left(\mu_{\alpha }-{\tilde{b}}^{2}\;\mu_{\alpha }^{-1}\right)$ for $\alpha =1,\ldots ,N_{S}$ . Given the specific structure of the coupling, one further has that $\mu_{\beta }=\gamma_{\beta }-\tilde{b}$ where *γ*_*β*_ are the eigenvalues of the Jacobian in each of the layers when removing the inter-layer coupling. Assuming that the noise amplitudes as well as the correlation times are the same in both components, one can rewrite Eq. 10 as,

$$\left\langle x_{i}^{2}\right\rangle =\eta_{0}^{2}\overset{N_{S}}{\underset{\alpha =2}{\sum }}\frac{v_{\alpha ,i}^{2}\left(\mu_{\alpha }^{2}+{\tilde{b}}^{2}\right)}{\left(\mu_{\alpha }^{2}-{\tilde{b}}^{2}\right)\left(\mu_{\alpha }^{2}-{\tilde{b}}^{2}-\mu_{\alpha }\tau^{-1}\right)}.$$ (16)

One can also calculate the variance when there is no timescale separation by remarking that the eigenmodes of the full Jacobian Eq. 5 are given by ${\left[v_{\alpha },\pm v_{\alpha }\right]}^{⊤}/\sqrt{2}$ with corresponding eigenvalues $\left(\mu_{\alpha }\pm \tilde{b}\right)$ for $\alpha =1,\ldots ,N_{S}$ . Using Eq. D1 in the Supplementary Material, the variance then reads,

$$\begin{array}{ll} \left\langle x_{i}^{2}\right\rangle & =\eta_{0}^{2}\overset{N_{S}}{\underset{\alpha =3}{\sum }}\frac{v_{\alpha ,i}^{2}\left(-\mu_{\alpha }\tau^{-1}+\mu_{\alpha }^{2}+{\tilde{b}}^{2}\right)}{\left(\mu_{\alpha }^{2}-{\tilde{b}}^{2}\right)\left(\tau^{-2}-2\mu_{\alpha }\tau^{-1}-{\tilde{b}}^{2}+\mu_{\alpha }^{2}\right)}\\ & \;+\frac{\eta_{0}^{2}{\left(N_{S}+N_{F}\right)}^{-1}}{2\tilde{b}\left(\tau^{-1}+2\tilde{b}\right)}. \end{array}$$ (17)

While Eqs 16, 17 are different in general, they become similar when one takes the two limits of short and long correlation times. In particular, in the long correlation time limit, they only differ by a constant term given by the second term in the right-hand side of Eq. 17. We therefore expect similar variances in the cases with and without timescale separation.

In the following section, we illustrate and confirm numerically the results discussed so far.

## 3 Numerical results

Here, we first numerically confirm the theory by analyzing a network where the oscillators in the fast and slow components are randomly chosen. Then, we illustrate the theory for the specific structures discussed in Section 2.

### 3.1 Fast and slow components randomly chosen

**FIGURE 5:**
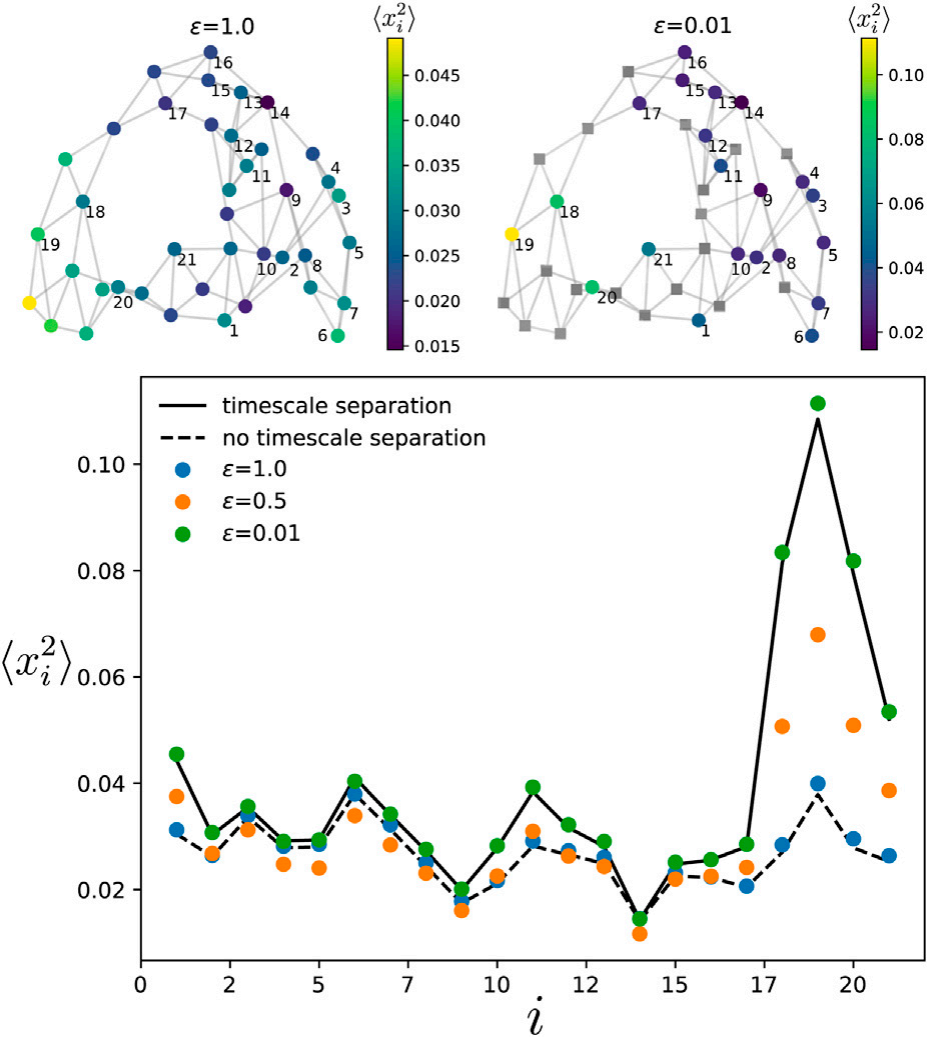
Variance of the phases in the slow component for the Watts-Strogatz network, with $N_{S}+N_{F}=40$ nodes $\left(N_{F}=19\right)$ , *p*_rewiring_ = 0.1 and four initial nearest neighbors, shown in the two top panels. The oscillators in the fast component are depicted by grey squares in the top right panel. In the lower panel, different colors for the dots correspond to different values of the timescale parameter *ϵ*. The dashed and solid black lines give the theoretical prediction, respectively, when there is no timescale separation (*ϵ* =1.0) and when there is a timescale separation (*ϵ* → 0). The variance depicted by the dots is obtained by time-evolving Eq. 1 over a single run. The noise correlation time is set to $\tau_{S}=\tau_{F}=0.5/\underline{d}$ , and amplitude to $\eta_{0}//\underline{d}=0.4$ . The natural frequencies of the oscillators satisfy var [*ω*_*i*_]=0.01.

We start by checking the analytical prediction when the oscillators in both components are randomly chosen. In Figure 5, we consider a Watts-Strogatz network (Newman, 2018) with *m* = 4 initial nearest neighbors and a rewiring probability *p*_rewiring_ = 0.1, of size $N_{S}+N_{F}=40$ , with respectively $N_{S}=21$ and $N_{F}=19$ oscillators in each component. The timescale separation is numerically simulated by taking the limit $\epsilon =\bar{d}/\underline{d}\ll 1$ . In Figure 5, we show the variance of the oscillators belonging to S for different values of 0 < *ϵ* ≤ 1. One observes that, for *ϵ* = 1, i.e., no timescale separation such that all the oscillators belong to S , the variance follows the analytical prediction of Eq. D1 in the Supplementary Material. Note that we are only showing the oscillators that belongs the slow component when *ϵ* < 1. When *ϵ* = 0.5, the variance differ from both prediction Eqs. (D1), (10). Eventually, for *ϵ* = 0.01, the numerical simulations follow the analytical prediction of Eq. 10, which corresponds to a system with a timescale separation. Besides confirming the theoretical prediction, Figure 5 shows that a timescale separation can induce significant changes in the variance of the oscillators in the slow component. Indeed, the variance of oscillator 19 when *ϵ* = 1 is approximately three times smaller than when *ϵ* = 0.01.

### 3.2 Disconnected oscillators in the fast component

**FIGURE 6:**
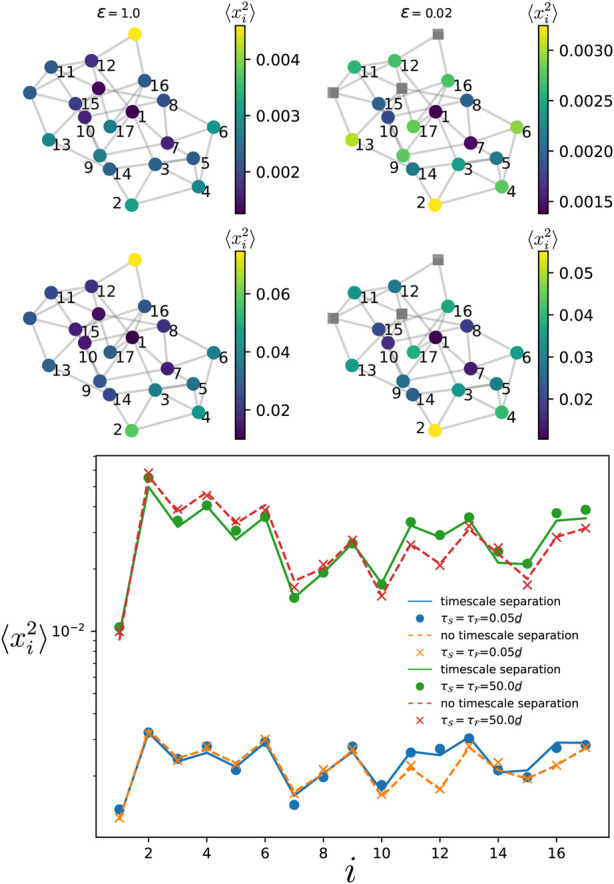
Variance of the phases in the slow component for the Watts-Strogatz network shown in the top panels, with $N_{S}+N_{F}=20$ nodes $\left(N_{F}=17\right)$ , *p*_rewiring_ = 0.3 and four nearest neighbors. The oscillators in the fast component are depicted by grey squares in the top right panels. The top panels correspond to the short correlation time limit while the middle ones correspond to the long correlation time limit. In the lower panel, the dashed and solid lines give the theoretical prediction, respectively, when there is no timescale separation (*ϵ* = 1.0) and when there is a timescale separation (*ϵ* → 0). The variance depicted by the dots and crosses is obtained by time-evolving Eq. 1 over a single run. The noise amplitude is set to $\eta_{0}/\underline{d}=0.4$ . The natural frequencies of the oscillators satisfy var [*ω*_*i*_] = 0.01.

We then move to the case described Section 2.4 where the oscillators in the fast component are disconnected. In Figure 6, we numerically simulate the dynamics of Eq. 1 on a Watts-Strogatz network with *m* = 4 initial nearest neighbors and a rewiring probability *p*_rewiring_ = 0.3, of size $N_{S}+N_{F}=20$ , with respectively $N_{S}=17$ and $N_{F}=3$ oscillators in each component. For the oscillators in the fast component, we picked the two oscillators with highest and lowest degrees, plus an additional one with an intermediate degree. As expected, we find that the effect of the timescale separation is limited to the oscillators directly connected to those in the fast compoenent. Overall, compared to the case without any timescale separation, the variance is not much affected.

### 3.3 All-to-all coupling from fast to slow component

**FIGURE 7:**
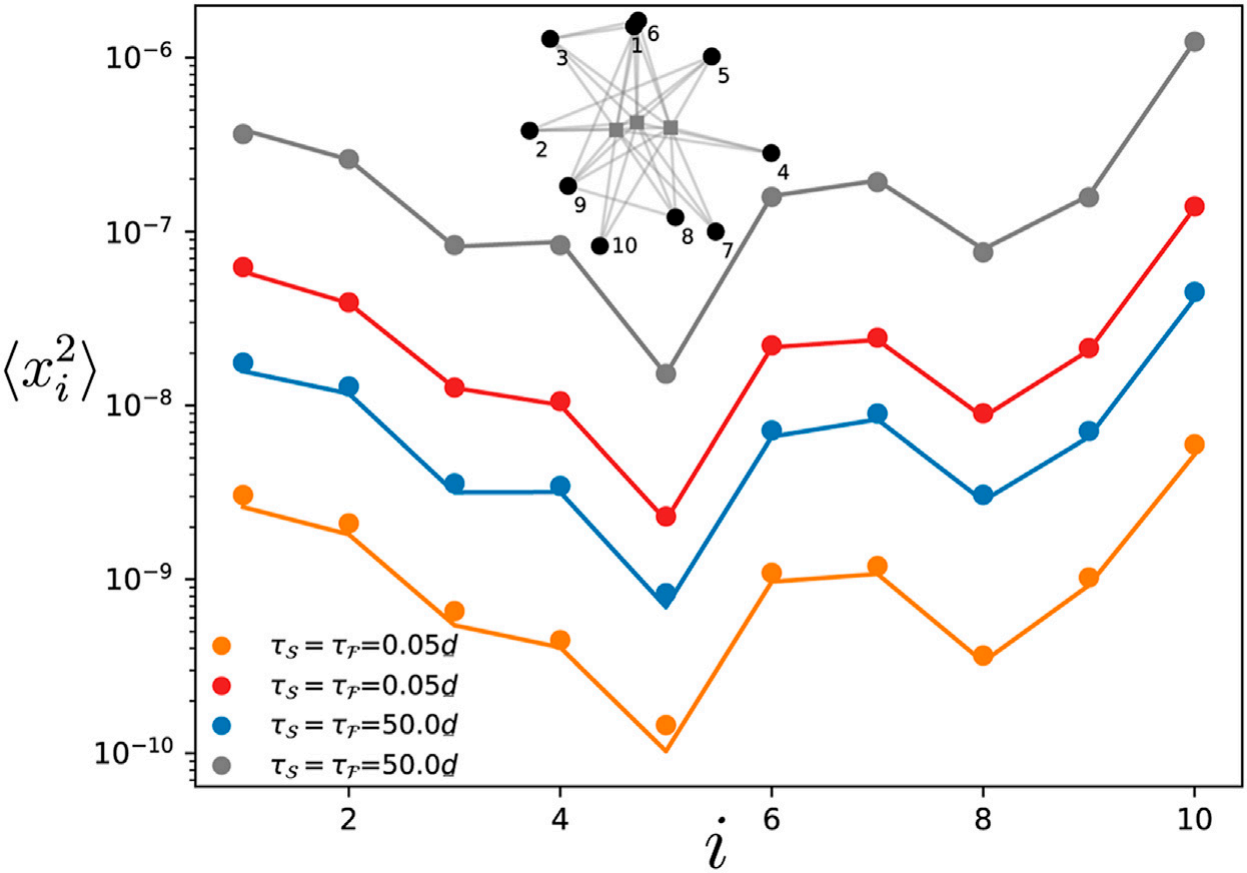
Variance of the phase deviations at every oscillator in the slow component. The variance is obtained by time-evolving Eq. 1 for a modified Erdős-Rényi network of 13 oscillators where three oscillators in the fast component are connected to all the oscillators in the slow component (see inset). The ratio of the damping parameters in the slow and fast component is $\bar{d}/\underline{d}=0.01$ . The natural frequencies are such that var [*ω*] = 0.41 for the orange and blue points and var [*ω*]=1.64 for the red and grey ones. Each dot and cross is obtained by time-averaging the variance over a single simulation of the dynamics while the solid lines give the theory Eq. 10. The noise amplitude is $\eta_{0,F}/\underline{d}=0.1$ .

We consider the situation described in Section 2.4.2 where oscillators in the fast component that have some connections to the slow ones, are connected to all of them. The numerical results are shown on Figure 7 where this particular setting has been simulated for a modified Erdős-Rényi network of $N_{S}+N_{F}=13$ oscillators with various correlation times of the noise and different levels of heterogeneity in the natural frequencies. Here, only the fast component is subjected to noise, while the slow component is noiseless. One observes that increasing the heterogeneity in the natural frequencies of the oscillators induces larger variances for the phase deviations. Not shown on Figure 7 is the homogeneous case of oscillators with identical natural frequencies, for which the variance vanishes as predicted in Section 2.4.2. While the heterogeneity increases the noise transmission from the fast to the slow component, one observes that the amplitude of the deviations is still rather small in Figure 7. Besides $u_{\alpha }^{⊤}J_{SF}$ being small, this is because the oscillators in the fast component with inter-component connections have relatively large degrees, which directly reduces the noise transmission in $\Gamma_{\alpha \beta }=u_{\alpha }^{⊤}J_{SF}J_{FF}^{-2}J_{FS}u_{\beta }$ .

### 3.4 All-to-all from slow to fast component

**FIGURE 8:**
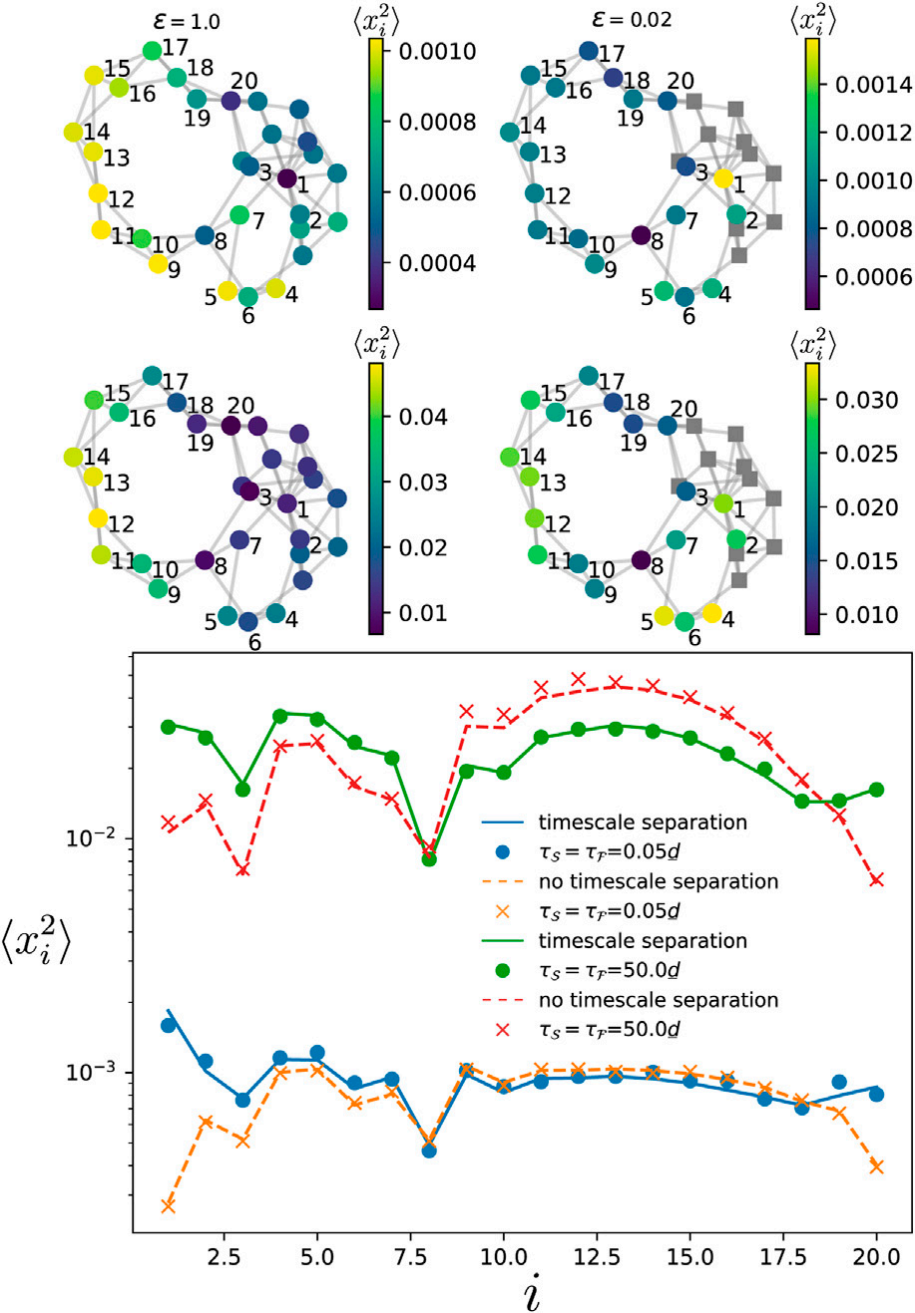
Variance of the phase deviations at every oscillator in the slow component when there is a timescale separation (right panels) and when there is no timescale separation (left panels), for a modified Watts-Strogatz network of $N_{S}+N_{F}=30$ where node one is connected to all the oscillators in the fast component. The oscillators depicted by grey squares are in the fast component while all the others are in the slow component. The top panels correspond to the short correlation time limit while the middle ones correspond to the long correlation time limit. The variance depicted by the dots and crosses is numerically obtained by time-averaging over a single realization of the dynamics Eq. 1. In the lower panel, the dashed and solid lines give the theoretical prediction, respectively, when there is no timescale separation (*ϵ* = 1.0) and when there is a timescale separation (*ϵ* → 0). The natural frequencies are such that var [*ω*] = 0.03. The noise amplitude is $\eta_{0,F}/\underline{d}=0.2$ .

**FIGURE 9:**
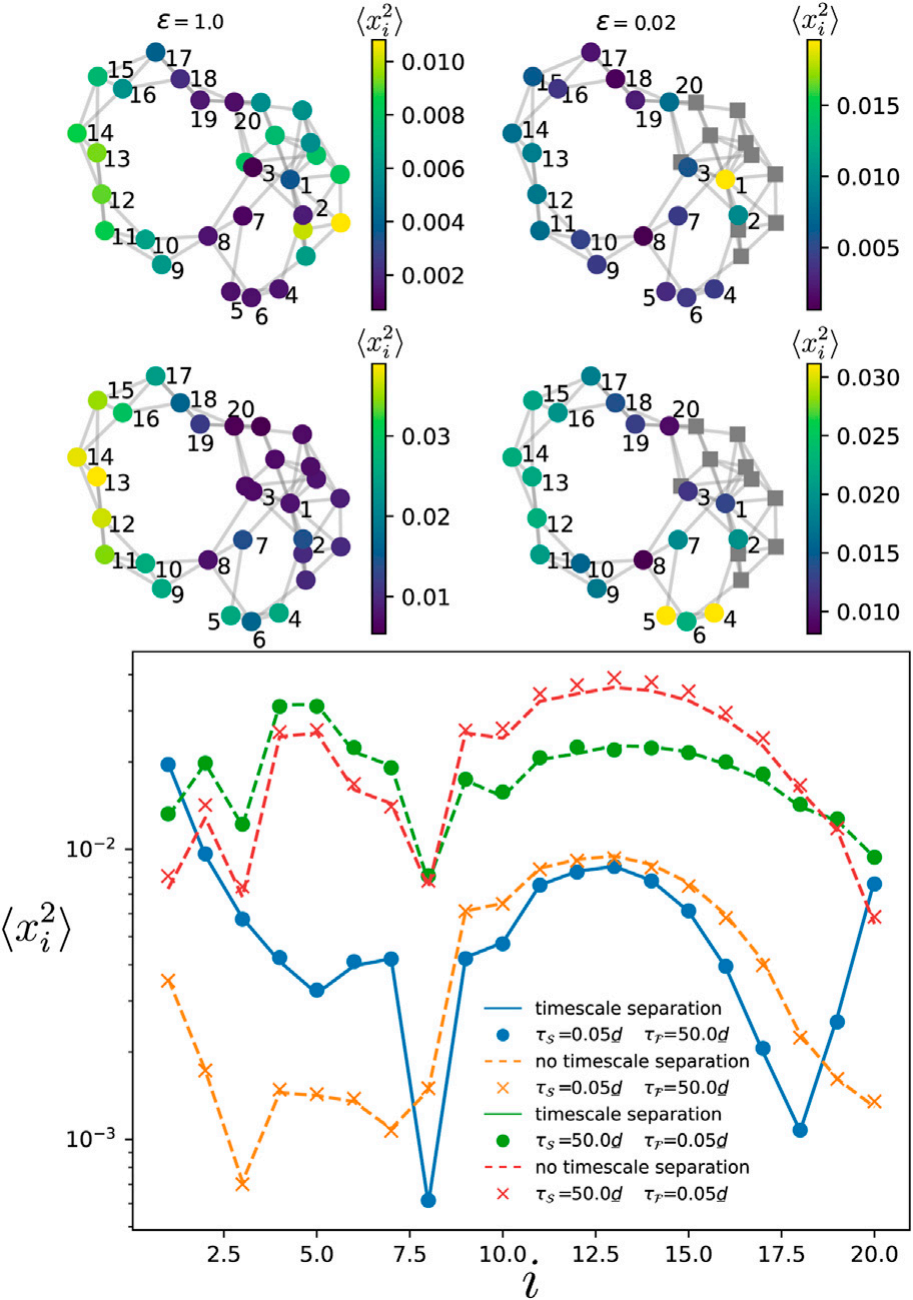
Variance of the phase deviations at every oscillator in the slow component when there is a timescale separation (right panels) and when there is no timescale separation (left panels), for a modified Watts-Strogatz network of $N_{S}+N_{F}=30$ where node one is connected to all the oscillators in the fast component. The oscillators depicted by grey squares are in the fast component while all the others are in the slow component. The top panels correspond $\lambda_{N_{S}}\tau_{F}\gg 1$ and $\lambda_{2}\tau_{S}\ll 1$ , and the middle panels to $\lambda_{N_{S}}\tau_{F}\ll 1$ and $\lambda_{2}\tau_{S}\gg 1$ . The variance depicted by the dots and crosses is numerically obtained by time-averaging over a single realization of the dynamics Eq. 1. In the lower panel, the dashed and solid lines give the theoretical prediction, respectively, when there is no timescale separation (*ϵ* = 1.0) and when there is a timescale separation (*ϵ* →0). The natural frequencies are such that var [*ω*] = 0.03. The noise amplitude is $\eta_{0,F}/\underline{d}=0.2$ .

In the other situation where some oscillators in the slow components are connected to a large fraction of the oscillators in the fast component, we showed in Section 2.4.3 that their variance is more important than oscillators with fewer or no connection to the fast component. This result is particularly interesting and intriguing, as in the regular situation where there is no timescale separation, oscillators that have a larger number of connections to the other elements typically have a smaller variance (Tyloo et al., 2019). Indeed, this is first illustrated in Figure 8 where the variance of each oscillator is given by the color map when there is no timescale separation (left top panels), and when there is a timescale separation (right top panels). In both correlation time limits (with $\tau_{S}=\tau_{F}$ ), one observes that oscillators belonging to S having smaller variances in the left top and middle panels are among the ones with larger variances in the right top and middle panels. In particular for the long correlation time limit, one also observes that the timescale separation modifies the variance of the oscillators far from the fast component. The theory and the numerical simulations for the two systems with and without timescale separation are confirmed in the bottom panel of Figure 8. We then move to the case where the noise correlation times are different in each component. In Figure 9, we show both limits $\lambda_{N_{S}}\tau_{F}\gg 1$ and $\lambda_{2}\tau_{S}\ll 1$ (top panels), and $\lambda_{N_{S}}\tau_{F}\ll 1$ and $\lambda_{2}\tau_{S}\gg 1$ (middle panels). Similar amplification of the fluctuations as in the previous case are observed. However, comparing the left and right middle panels, one remarks that some oscillators well connected to the fast component keep rather small variances while others having fewer connections become more vulnerable.

### 3.5 Layered networks

**FIGURE 10:**
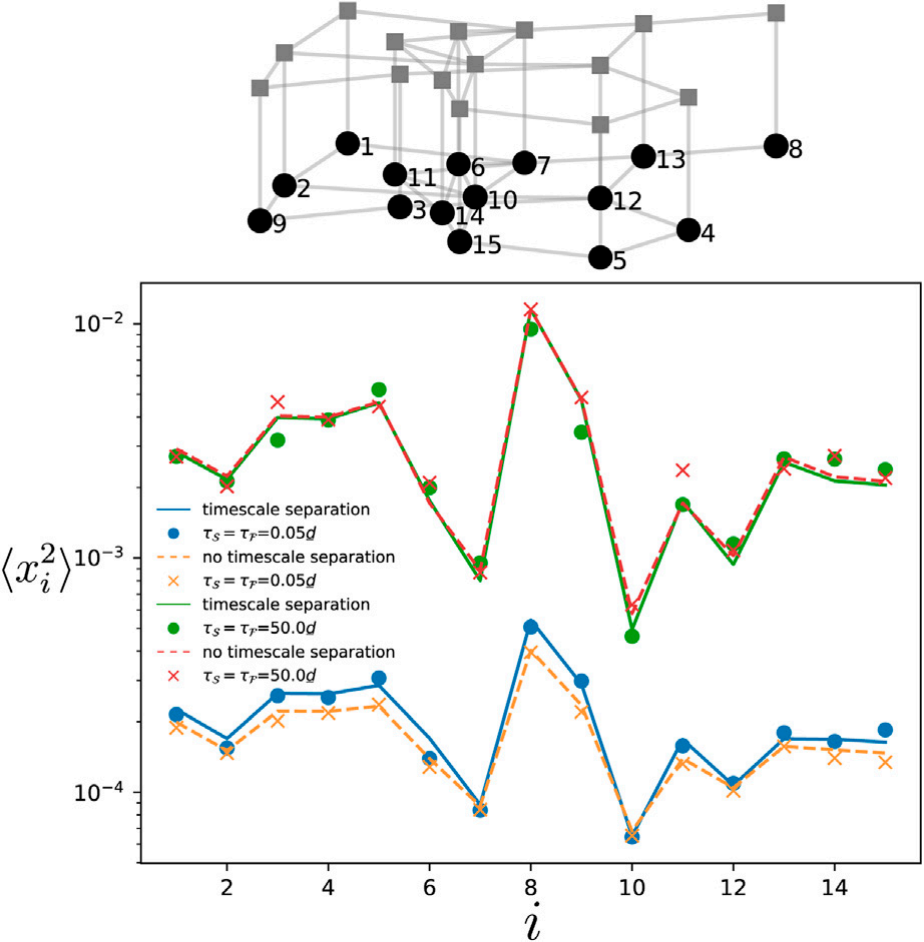
Variance of the phase deviations at every oscillator in the slow component. The variance is obtained by time-evolving Eq. 1 for a layered network made of two copies of an Erdős-Rényi network of 15 oscillators with edge probability 0.28 (see top panel). One layer is the fast component while the other is the slow one as depicted in the above network. The natural frequencies are identical and vanishing for all oscillators. Each dot and cross is obtained by time-averaging the variance over a single simulation of the dynamics. The solid and dashed curves give respectively, the theory Eq. 10 when there is no timescale separation (*ϵ* = 1), and when there is a timescale separation (*ϵ* =0.01). The noise amplitude is $\eta_{0,F}/\underline{d}=0.1$ .

Here, we check the theory when the system is defined on a layered network, i.e., the slow and fast components each corresponds to one layer. We consider the specific setting where both layers have the same network connectivity and the inter-layer coupling is made through single connections between corresponding oscillators in each component (such structure are sometimes called *multiplex* (Newman, 2018)). In Figure 10, the numerical simulations for the variance (dots and crosses) match the theory Eqs. 10 and Eq. D1 in the Supplementary Material (solid and dashed lines) for various correlation times of the noise (homogeneous, i.e., $\tau_{F}=\tau_{S}$ ). The blue and orange data points correspond to the situation where there is a timescale separation between the two components, while for the red and green ones, there is no timescale separation. Interestingly, one observes that the two different situations produce similar variances for the phases. As predicted in Section 2.4.4, in the limit where *τ* is the longest timescale in the system, the variances in the two situations are close to each other (green and red data points in Figure 10). A similar effect is observed in the short correlation time limit, where the variance is only marginally impacted by the timescale separation. We do not show but found similar behavior in the case of heterogeneous noise correaltion time in the two components. This indicates that networks having a layered structure are robust to timescale separation when the slow and fast components are defined on each layer.

## 4 Conclusion

Physiological systems are composed of a multitude of synchronized dynamical units evolving on various timescales. It is therefore relevant to investigate how these different timescales impact the synchronization dynamics of networked phase oscillators. Here, we considered networks of synchronized phase oscillators where a timescale separation divides the units into a slow and a fast component. Using Mori-Zwanzig formalism, we derived a reduced dynamical system describing the time-evolution of the slow component. We used the latter to assess the resilience of the slow component by calculating the variance of the phase deviations. We obtained a closed-form expression for the variance of each oscillator as a function of the eigenmodes of the reduced Jacobian. Interestingly, noise propagation from the fast to the slow component essentially depends on the mixing of the different eigenmodes. The precise mixing is given by the inter- and intra-component coupling structures. In particular, we showed that oscillators that have a small variance when there is no timescale separation, might have a strongly amplified variance when there is a timescale separation and they have numerous connections to the fast component. Also, we found that when the fast and slow components are connected over a layered structure, the variance of the oscillators is mostly insensitive to a timescale separation. When oscillators in the fast component are disconnected, the effect of the timescale separation remains local.

The theory presented here highlights the importance of timescales to assess the resilience of coupled phase oscillators. Some oscillators that might be the most robust within one ratio of the timescales, might become the most fragile ones for another ratio (see Figure 8, 9).

While the results of this manuscript were obtained for Kuramoto oscillators, they apply more generally to coupled dynamical system evolving close to a stable fixed point, so that the linear approximation is valid. For example, one could use the same framework to investigate coupled dynamical systems with adaptive coupling strength close to a stable fixed point. In this case, the linearization Eq. 4 will include the dynamics of the coupling strength. One can then choose which variables will undergo a timescale separation, i.e., which variables belong to the slow and fast components.

## 5 Future work

The present manuscript focused on a single timescale separation where the oscillators are separated into a slow and a fast component. Future research should consider more than one timescale separation to model more precisely dynamics such as physiological networks, as described in the introduction. Also, we focused here on the slow component, but one should evaluate the resilience of the fast component as well. Another assumption of these results, is that the noise is small enough such that we can only consider the small fluctuation of the slow component. One could consider transition between basins of attraction, and evaluate how the rate of large deviations is affected by the timescale separation.

## Data Availability

The original contributions presented in the study are included in the article/Supplementary Material, further inquiries can be directed to the corresponding author.
