# Supplementary material for: Resilience of the slow component in timescale-separated synchronized oscillators
Source: Front Netw Physiol. 2024 Jun 19;4:1399352. doi: 10.3389/fnetp.2024.1399352 (PMC11220911; doi:10.3389/fnetp.2024.1399352)
Supplement: Supplementary file 1 [file Presentation1.pdf]

Supplementary Material for: Resilience of the slow component in timescale separated synchronized oscillators

Melvyn Tyloo
Theoretical Division and Center for Nonlinear Studies (CNLS), Los Alamos National Laboratory, Los Alamos, NM 87545, USA

(Dated: May 10, 2024)

Appendix A: Overview of Mori-Zwanzig formalism

The idea behind the Mori-Zwanzig formalism is to obtain the time-evolution of the resolved variables in the system, which represents only a subset of the total variables. Let us consider, as an example, the following linear system,

$$\dot{\mathbf{x}} = \mathbf{A}\mathbf{x}\,, \tag{A1}$$

where the variables are separated into a resolved $\mathbf{x}_{\rm rev}$, and an unresolved part $\mathbf{x}_{\rm unr}$ as, $\mathbf{x} = (\mathbf{x}_{\rm res}\,, \mathbf{x}_{\rm unr})^\top$, such that,

$$\begin{bmatrix}\dot{\mathbf{x}}_{\rm res}\\ \dot{\mathbf{x}}_{\rm unr}\end{bmatrix} = \begin{bmatrix}\mathbf{A}_{11} & \mathbf{A}_{12}\\ \mathbf{A}_{21} & \mathbf{A}_{22}\end{bmatrix}\begin{bmatrix}\mathbf{x}_{\rm res}\\ \mathbf{x}_{\rm unr}\end{bmatrix}. \tag{A2}$$

One can then write the unresolved part as,

$$\mathbf{x}_{\rm unr}(t) = e^{\mathbf{A}_{22}t}\,\mathbf{x}_{\rm unr}^{(0)} + \int_0^t e^{\mathbf{A}_{22}(t-t')}\,\mathbf{A}_{21}\mathbf{x}_{\rm res}(t'){\rm d}t'\,, \tag{A3}$$

where $\mathbf{x}_{\rm unr}^{(0)}$ denotes the initial condition. Then, to obtain the time-evolution of the resolved variables, one can plug Eq. (A3) into the first row of Eq. (A2) , such that,

$$\dot{\mathbf{x}}_{\rm res}(t) = \mathbf{A}_{11}\mathbf{x}_{\rm res}(t) + \mathbf{f}(t) + \int_0^t \mathbf{K}(t-t')\mathbf{x}_{\rm res}(t'){\rm d}t'\,, \tag{A4}$$

where $\mathbf{f}(t) = \mathbf{A}_{12}e^{\mathbf{A}_{22}t}\,\mathbf{x}_{\rm unr}^{(0)}$ and $\mathbf{K}(t-t') = \mathbf{A}_{12}e^{\mathbf{A}_{22}(t-t')}\,\mathbf{A}_{21}$. In the situation considered in the main text, the memory kernel $\mathbf{K}(t-t')$ can be written down explicitly. Moreover, using the timescale separation, one can also calculate the integral. A good introduction is given in [1] .

Appendix B: Convergence to a Dirac-δ distribution

To obtain Eq. (7) from Eq. (6) , we let $\epsilon \to 0$. Doing so, a Dirac-δ appears in Eq. (6) as,

$$\lim_{\epsilon\to 0}\int_0^t \epsilon^{-1}e^{-ct'/\epsilon}f(t'){\rm d}t' = \tag{B1}$$

$$\lim_{\epsilon\to 0}[-e^{-ct/\epsilon}f(t)+f(0)]c^{-1} + \int_0^t \lim_{\epsilon\to 0} e^{-ct'/\epsilon}f'(t')c^{-1}{\rm d}t'$$
$$= f(0)c^{-1}\,,$$

where $c>0$ and we used an integration by parts in the first equality and the dominated convergence theorem. Using this equality, one recovers the result of Eq. (7) .

Appendix C: Details to obtain Eq. (10)

One can calculate the long-time limit of the variance of the deviations x_i from the synchronized state using the part of Eq. (9) that is orthogonal to $\mathbf{u}_1$. One then has,

$$\langle x_i^2\rangle = \lim_{t\to\infty}\sum_{\alpha,\beta=2}^{N_\mathcal{S}}\langle c_\alpha c_\beta\rangle u_{\alpha,i}u_{\beta,i} \tag{C1}$$

$$= \lim_{t\to\infty}\sum_{\alpha,\beta=2}^{N_\mathcal{S}}\int_0^t\int_0^t {\rm dt}_1{\rm dt}_2 e^{\lambda_\alpha(t-t_1)}e^{\lambda_\beta(t-t_2)} \tag{C2}$$
$$\times\sum_{j,k=1}^{N_\mathcal{S}}\langle\xi_j(t_1)\xi_k(t_2)\rangle u_{\alpha,j}u_{\beta,k}u_{\alpha,i}u_{\beta,i}\,.$$

The two-point correlator of the noise satisfies $\langle\xi_j(t_1)\xi_k(t_2)\rangle = \eta_0^2\,\delta_{jk}\exp(-|t_1-t_2|/\tau_\mathcal{S}) + \eta_0^2\,[\mathbf{J}_{\mathcal{S}\mathcal{F}}\mathbf{J}_{\mathcal{F}\mathcal{F}}^{-2}\mathbf{J}_{\mathcal{F}\mathcal{S}}]_{jk}\exp(-|t_1-t_2|/\tau_\mathcal{F})$. Using the latter relation and after some algebra, one obtains the variance in the slow component Eq. (10) .

Appendix D: No timescale separation

One can also calculate the variance of the oscillators belonging to $\mathcal{S}$ (and also $\mathcal{F}$) when there is no timescale separation. Assuming as previously that the oscillators in $\mathcal{S}$ and $\mathcal{F}$ are subject to noises with correlation times $\tau_\mathcal{S}$ and $\tau_\mathcal{F}$ respectively, and that the standard deviations are homogeneous, one has,

$$\langle x_i^2\rangle = \eta_0^2\sum_{\alpha,\beta=2}^{N_\mathcal{S}+N_\mathcal{F}}\sum_{j\in\mathcal{S}}\frac{(\gamma_\alpha+\gamma_\beta-2\tau_\mathcal{S}^{-1})q_{\alpha,j}q_{\beta,j}q_{\alpha,i}q_{\beta,i}}{(\gamma_\alpha+\gamma_\beta)(\tau_\mathcal{S}^{-1}-\gamma_\alpha)(\tau_\mathcal{S}^{-1}-\gamma_\beta)} \tag{D1}$$
$$+\,\eta_0^2\sum_{\alpha,\beta=2}^{N_\mathcal{S}+N_\mathcal{F}}\sum_{j\in\mathcal{F}}\frac{(\gamma_\alpha+\gamma_\beta-2\tau_\mathcal{F}^{-1})q_{\alpha,j}q_{\beta,j}q_{\alpha,i}q_{\beta,i}}{(\gamma_\alpha+\gamma_\beta)(\tau_\mathcal{F}^{-1}-\gamma_\alpha)(\tau_\mathcal{F}^{-1}-\gamma_\beta)}\,,$$

for $i = 1,...,N$, where $\mathbf{q}_\alpha$ are the eigenvectors of the Jacobian Eq. (5) , with corresponding eigenvalues γ_α .

[1] F. Caravelli and Y. T. Lin, arXiv preprint arXiv:2308.13653 (2023).
